# Who cares for happy cows? Exploring views of dairy stakeholders around an imaginary automated animal-based welfare assessment tool

**DOI:** 10.3389/fvets.2025.1704112

**Published:** 2025-12-04

**Authors:** Lena-Mari Tamminen, Niclas Högberg, Karin Berggren, Louise Winblad von Walter, Gabriela Olmos Antillón

**Affiliations:** 1 Veterinary Epidemiological Unit, Department of Clinical Sciences, Swedish University of Agricultural Sciences, Uppsala, Sweden; 2 Växa Sverige, Stockholm, Sweden

**Keywords:** dairy cattle, future of livestock farming, management and husbandry, animal welfare, sensors, UX, PLF

## Abstract

**Introduction:**

Precision Livestock Farming (PLF) provides additional opportunities beyond focusing solely on production and health-related traits, including the evaluation of animal welfare. This study examines the complexities of adopting an imaginary automated welfare assessment tool in Swedish dairy farming.

**Materials and methods:**

Through an iterative qualitative design, we engaged dairy farmers (*n* = 10), advisors (*n* = 5), dairy industry experts (*n* = 2), and PLF managers (*n* = 3) to co-develop insights and integrate multiple stakeholders’ perspectives. Online focus groups (*n* = 7) served as a platform to explore participants’ cultural nuances, discourses, and practical challenges surrounding animal welfare indicators related to feeding, comfort, health, and complementary behaviours.

**Results:**

A reflexive thematic analysis exposes strains between farmers’ perceptions and other stakeholders’ meanings and practices. Despite other stakeholders’ assumptions of ‘farm blindness,’ farmers demonstrate awareness and interest in comfort-related welfare indicators. However, they experience difficulties implementing changes due to limited agency and infrastructural capacities. Other stakeholders often interpreted the lack of farmers’ actions as indifference, overlooking farmers’ nuanced prioritisation strategies. Equally, conversations underscore farmers’ doubts about the commitment and backing of supervising bodies as they hesitate regarding data sharing among interested stakeholders. Crucially, this highlights a lack of shared understanding and motivation for the welfare assessment, and a lack of long-term advisory support that aligns with farmers’ capabilities and constraints.

**Conclusion:**

Our study underlines the importance of bridging the gap between scientific knowledge and on-farm practices, particularly in defining actionable guidelines for addressing welfare concerns. Regardless of the concretion of our imaginary automated tool, we conclude that stakeholders could readily foster greater engagement with animal welfare issues by recognising farmers’ agency capabilities and providing tailored, contextually relevant support that signals the industry’s support to farmers, not only a self-need to retain a delicate license to operate thus truly facilitating meaningful gains in animal welfare.

## 1 Introduction

Precision livestock farming (PLF) and digital tools have emerged as powerful aids in dairy cattle management, primarily focusing on detecting and managing health-related issues (1–4). These systems, often centered around accelerometers, sensors, and automated data collection, have been extensively applied to monitor disease, detect estrus, and optimise milk production (1). However, while health is a fundamental component of animal welfare, current solutions prioritise illness identification over a holistic view of welfare that encompasses behavioural expression, positive experiences, and overall quality of life (5). This gap reflects a broader challenge in dairy farming: the need for welfare monitoring that extends beyond disease detection to include an integrated understanding of cows’ lived experiences.

Although PLF technologies are developing quickly, evidence on their fit with farmers’ priorities and with the requirements of other stakeholders who rely on PLF-derived data is still emerging (6–8). Nonetheless, the uptake and success of automated welfare monitoring systems depend on their technical accuracy and how they fit within daily routines, resource constraints, and end-user values and needs (4, 9). While welfare certification schemes and benchmarking are increasingly relevant in current market-driven dairy production, farmers often perceive these assessments as burdensome and complex to implement (10–12). Moreover, the concept of “welfare” itself is socially constructed, carrying different meanings across geographies and interacting actors, including farmers (key end-users), veterinarians, advisors, and governance bodies, each interacting with PLF and digital tools in distinct ways (10) and thus being impacted differently. This means that digitalisation’s perceived positive impacts might not meet expectations.

Given these complexities, the co-development of digital welfare assessment tools is critical. Existing welfare monitoring protocols remain manual, time-consuming, infrequent, and subjective to assessor skills and re-calibration, making them sub-optimal for real-time farm decision-making (1, 13, 14). Yet, these are seen by other actors (e.g., governance bodies and milk processors) as essential measures for production quality and transparency. Automating welfare assessments requires thoughtful and detailed technical validation and a nuanced understanding of how end-users—farmers and their advisors interpret, utilise, and respond to welfare data (15). Without such insights, even the most sophisticated welfare monitoring systems risk being underutilised or misunderstood.

Understanding how automated welfare assessments can be taken up in practice requires not only technical validation but attention to implementation processes. Implementation theory shows that new practices succeed when they are workable and meaningful for those involved. For instance, the COM-B model of behaviour change (16) proposes that change occurs when capability, opportunity, and motivation align. Similarly, Normalization Process Theory (13) emphasises the collective sense-making and integration work needed for new routines to become embedded. These perspectives have rarely been applied in farm animal welfare assessment research, yet they are valuable lenses for analysing why digital welfare tools may or may not fit into daily dairy farming.

Our project, therefore, aimed to bridge this gap by exploring the perspectives and experiences of dairy farmers regarding welfare monitoring, including automated solutions.

## 2 Materials and methods

### 2.1 Study design and participant selection

Our study explored the meanings, tensions, and practical challenges associated with welfare assessments in real-world farm settings through an iterative, qualitative approach. Three rounds of discussions with seven stable (i.e., members of the groups did no change across sessions) focus groups (FG) were conducted with Swedish dairy farmers (*n* = 10) and other dairy industry stakeholders, veterinarians specialised in dairy health and welfare (*n* = 2), PLF product managers (*n* = 3) and dairy advisors (*n* = 5). The discussion topics for each session were developed iteratively based on previous internal discussions within the Visionary Welfare Assessment project steering committee, which included diverse stakeholders of the dairy sector (veterinarians, farmers, dairy health advisors, milking machine developers), animal welfare, machine learning and social scientists. The selection of participants aimed to encompass diverse perspectives on animal welfare assessment, automation, and its practical implications in dairy farming. Participants were recruited through professional networks, industry events, and existing research collaborations. To ensure a broad representation of Sweden’s farming conditions, farmers were selected based on farm size, geographical location, and adoption of PLF technology. Dairy advisors and industry representatives were included to provide expertise on welfare assessment, implementation challenges, and sector-wide considerations.

### 2.2 Focus group procedure

We structured the focus groups into three iterative sessions. All focus group sessions were conducted between December 2023 and March 2024. In the first session, we explored the meaning and relevance of animal welfare, aiming to establish a baseline understanding of how participants perceive animal welfare, how assessments impact daily farm practices, and to surface any tensions surrounding their implementation. During the discussions, we equally examined how welfare assessment tools integrate into daily decision-making and farm management.

In the second session, we examined the level of integration of precision livestock farming (PLF) technologies into animal welfare assessment and their influence on decision-making as experienced by the different stakeholders. We encouraged participants to discuss their needs for automated monitoring, the benefits they anticipated, concerns about technology adoption, and barriers to implementation. These discussions centered on four welfare areas inspected in Sweden: feeding and nutrition, animal comfort, health, and complementary behaviours—as well as the associated measures. We emphasised the influence of contextual factors such as farm routines, farmer identity, and current management practices.

In the third and final session, we addressed concerns regarding data access and usability to support the roles of farmers and other stakeholders.

We conducted all the sessions online to accommodate participants’ schedules, each lasting 90 min. Each session had specific guiding questions accessible via a Mentimeter presentation (17). Each Mentimeter block lasted 10–15 min and alternated with facilitated discussions to expand on participants’ reflections. The format facilitates the anonymous sharing of experiences among peers. In addition to verbal communication, each session had one facilitator (LMT or GOA) and a screen manager (GOA or KB) who shared and controlled the Mentimeter presentation. The facilitators used the presentation to guide the discussion and gather complementary views from participants interactively, aiding in anonymously gathering impressions from participants in selected questions. Moreover, depending on the session, there were up to three note-takers (KB, GOA or NH). Note-takers supported the facilitators in prompting questions for a more profound understanding or discussion. After each session, all authors who attended met to reflect on the insights and document key observations. All sessions were audio-recorded and transcribed verbatim.

The complete wording of all Mentimeter questions used in the three focus group sessions is available in Supplementary Material 1 and archived in the Open Science Framework repository.^1^

### 2.3 Data analysis

We conducted a Reflexive Thematic Analysis (RTA) (18–20) to interpret the qualitative dataset. The RTA framework was grounded in a constructivist epistemology, which assumes meaning as context-dependent and co-constructed between participants and the researchers. Thus, the researcher’s reflexivity is considered a valuable resource for interpretation within the RTA framework. The analysis was predominantly inductive, situated within an inductive-deductive continuum. Interviews were open-coded, emphasising the meanings participants assigned to animal welfare, its assessment, and the impact PLF technologies have on decision-making for the management of the herd and individual cows. The analysis went beyond a descriptive level, using our reflexivity to articulate underlying assumptions, beliefs, and structural factors that shape participants’ perspectives.

LMT and GOA were the primary authors responsible for the analysis. After independently immersing in the transcripts and post-session notes, we generated initial codes that mapped salient features across the dataset. An iterative discussion between LMT and GOA transformed these codes into provisional themes, which KB and NH critiqued for coherence and depth. The resulting thematic framework comprehensively accounts for participants’ core concerns and perspectives while remaining closely aligned with the study’s objectives.

Rankings done by participants in Mentimeter were summarised and visualised in R statistical software (21–23).

### 2.4 Author positionality statement

The research team combines expertise in animal welfare science, veterinary medicine, precision livestock farming, and qualitative research. LMT is a veterinary epidemiologist, with a background in veterinary medicine, focusing on biosecurity, animal health, welfare, production efficiency and resilience. NH has a background in veterinary medicine specialised in digitalisation and data-driven decision-making in animal production systems, focusing on integrating PLF in animal welfare monitoring. KB is a veterinarian currently doing her doctoral studies in veterinary epidemiology. She has experience in one health research. GOA is a veterinarian and applied animal-welfare researcher, trained in medical social science, whose work uses quantitative and qualitative methods to study on-farm and veterinary practices. Their combined expertise and interdisciplinary approach informed the study design, data collection, analysis, and interpretation of findings.

### 2.5 Ethical statement

In consultation with the Ethics and Legal Department at the Swedish University of Agricultural Sciences (SLU), and in agreement with the Swedish Ethical Authority, this study was determined not to require a special ethical provision or permit under Swedish law (SFS 2003:460). Nevertheless, the study adhered to the ethical guidelines established by the Swedish Research Council (41). Before participation, informed consent was obtained from all individuals, ensuring confidentiality and the pseudo-anonymisation of responses. No sensitive personal data was collected.

## 3 Results

Here we report the meanings and tensions participants attached to animal welfare meaning and its on-farm evaluation across the three focus group sessions, integrating qualitative analysis with the interactive ranking exercises during each session. Four interlinked themes organise the results. First, we show how “good welfare” is understood and operationalised in daily practice: both groups, Farmers (F) and other Stakeholders (S) coincide on the same domains (nutrition/feeding, health, comfort/behaviour), but their work/role-specific priorities lead farmers to favour indicators that cue immediate action, while the rest of the stakeholders lean toward system-level trends and benchmarking. Second, we examine why “the best welfare is worth it, but difficult to achieve”: structural constraints and labour/skill demands mean automation often saves time without automatically improving decisions, and where health indicators and related alarms are read mainly as detection cues. Such ideas lead us to the third theme, where we unpack what “automation” of welfare assessment means to different actors— work efficiency for farmers; standardisation, comparison and potential decision uplift for stakeholders—clarifying the split views seen in the relationship between decision-value and level of automation, comparing current vs. future desired scenarios. Finally, we address data access, context and trust, outlining when and why sharing beyond the farm adds value or risk. Read together, these themes explain the overarching pattern: shared beliefs about importance but diverging views on usefulness for improving day-to-day management and the consequences.

### 3.1 Theme 1: Happy cows every day: pride, practicality, and the indicators that count

Participants consistently framed good welfare as cows that are well, calm, and thriving. Farmers expressed pride in sustaining that state through attentive, everyday work and also reflected on how the happiness of their cows was connected to their own well-being. Their language is practical and affective at once, mentioning happy and secure animals that have what they need, in particular in relation to quick responses when something deviates.

*“I'm quite proud when I can show our cows and the barn to people who do not know what we’re doing. I can show them that our cows are happy so to speak. …. It is a lot more fun to work when you see that the cows are happy. That usually makes us happy too.”* (Farmer, FG-1)

*“We reasoned that good animal welfare is essentially happy animals… and also that sustainability and animal welfare go together to some extent.”* (Farmer, FG-4)

*“I wrote this: Cows that are feeling well and have everything they need… and then get appropriate treatment, or whatever else they need.”* (Farmer, FG-7)

**Figure 1:**
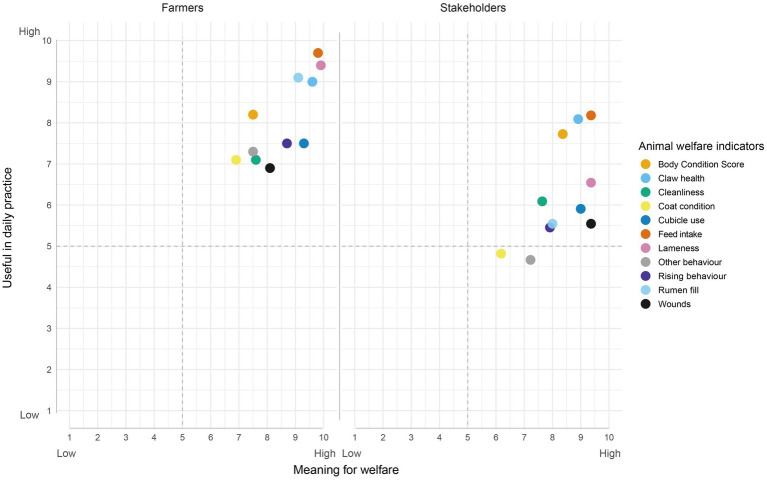
Average ranking of eleven animal welfare indicators according to their perceived meaning for animal welfare (x-axis) and their usefulness in daily practice (y-axis), split by group of participants. Right-hand side image: farmers’ ranking (*n* = 10), left-hand side image: other Stakeholders’ ranking (*n* = 10).

Figure 1 presents a summary of the ranking of 11 animal and resource-based measures used in Sweden to assess the welfare of a herd, acquired in the first session of each focus group. Participants scored the welfare indicators based on their usefulness in daily practice and meaning for animal welfare. Both groups rated all measures of high importance for animal welfare and there was a broad agreement across all stakeholders on which areas have the highest meaning for animal welfare: nutrition/feeding and health, in particular claw health and lameness. For usefulness in daily decisions, farmers overall tended to perceive a higher usefulness for all welfare indicators in their daily practice compared to other types of participants. This tendency may partly reflect how the question framed ‘usefulness in daily work,’ which naturally resonates more with farmers who manage cows daily than with stakeholders engaged at advisory or policy levels.

There were also interesting differences in which specific indicators farmers and stakeholders found most useful for their work. Farmers tended to rank animal welfare measures that change relatively quickly, i.e., indicators in which they can see changes daily. For example, in the area of feeding/nutrition farmers reported greater daily usefulness of rumen fill compared to body condition score which was highlighted as more useful by the stakeholders. In addition, while both groups ranked claw health as highly useful, farmers also ranked lameness, a direct measure of locomotion ability, higher than the stakeholders.

Also in discussions, farmers mentioned prioritising detecting deviations they can act on immediately, especially signs of compromised health. Indicators connected to immediate health deviations were perceived to have high usefulness as they cue near-term, controllable action. The farmers describe how they rely on digital lists to identify deviations but also emphasise the role of combining information from sensors with human observations to make decisions.

*“I check SenseHub every morning… I get an attention list and then go and check it as well, using the human eye.”* (Farmer, FG-4)

*“It’s similar to the top reports/lists… we follow them completely—even though we double-check manually*.” (Farmer, FG-3)

The focus on immediate solutions reflects farmers’ need for control as well as their preference for straightforward routines and practical fixes that solve problems and keep things running. *“For heat detection we use the simplest programme…and keep watching the animals all the time. They [referring to the staff] must learn to use their “djurögat!” [a Swedish term referring to having a pair of eyes on the cow and the ability to observe the cow status, a farmer-skill that comes with experience working with cows]*” (Farmer, FG-1).

Farmers emphasised that health-related alerts carry immediate relevance by signalling conditions directly affecting animal welfare and performance. Thus, acting promptly was seen as part of good stockmanship skills. Other stakeholders often work at a different level of the system, so they emphasise trends, comparison and communication—the kinds of indicators that help develop systems, benchmark performance, or create products for the market.

*“With this, especially at the system level, you must compare—sometimes even across species.”* (Stakeholder, FG-2)

*“The cow-calf idea is an example… a niche product that can find buyers—there’s a consumer dimension here*.” (Stakeholder, FG-2)

Participants*’* voices and the ratings show that welfare is valued and already operationalised in daily work, but the indicators people reach for depend on what job they are trying to do. The ranking of animal welfare measures together with the conversations, suggest that the perceived usefulness of indicators is not solely related to the perceived importance for animal welfare but equally reflects the needs of the respective working role. To one end farmers act on immediate management cues, while the rest lean towards data for trends, benchmarking or communication of animal welfare status.

### 3.2 Theme 2: The best welfare is worth it, but difficult to achieve

Farmers prioritise signals that flag fixable deviations now—often starting with health and feeding—while other stakeholders prioritise signals that build systems and narratives over time. This reflects where each group sits in the production system. These narratives set up the following idea: achieving “the best welfare” is worth it, yet complex without structural changes and support. Meaning that even when the proper signals are seen, turning them into sustained change is the harder step.

Participants agreed that high welfare is worth striving for, yet current tools are used foremost to detect problems—a stance that prioritises identification of sick animals and near-term action. Farmers described becoming more effective at finding issues with digital tools, in particular related to health problems.

*“Automation is fairly low across everything* [referring to the status of own farm]*… health was the one that was fairly high.”* (Farmer, FG-3)

*“We have a very good way to find cows not feeling well: drops in milk yield, milk temperature (heat), rumination & eating time… The system scores and ranks cows by deviation from normal.”* (Stakeholder, FG-7)

**Figure 2:**
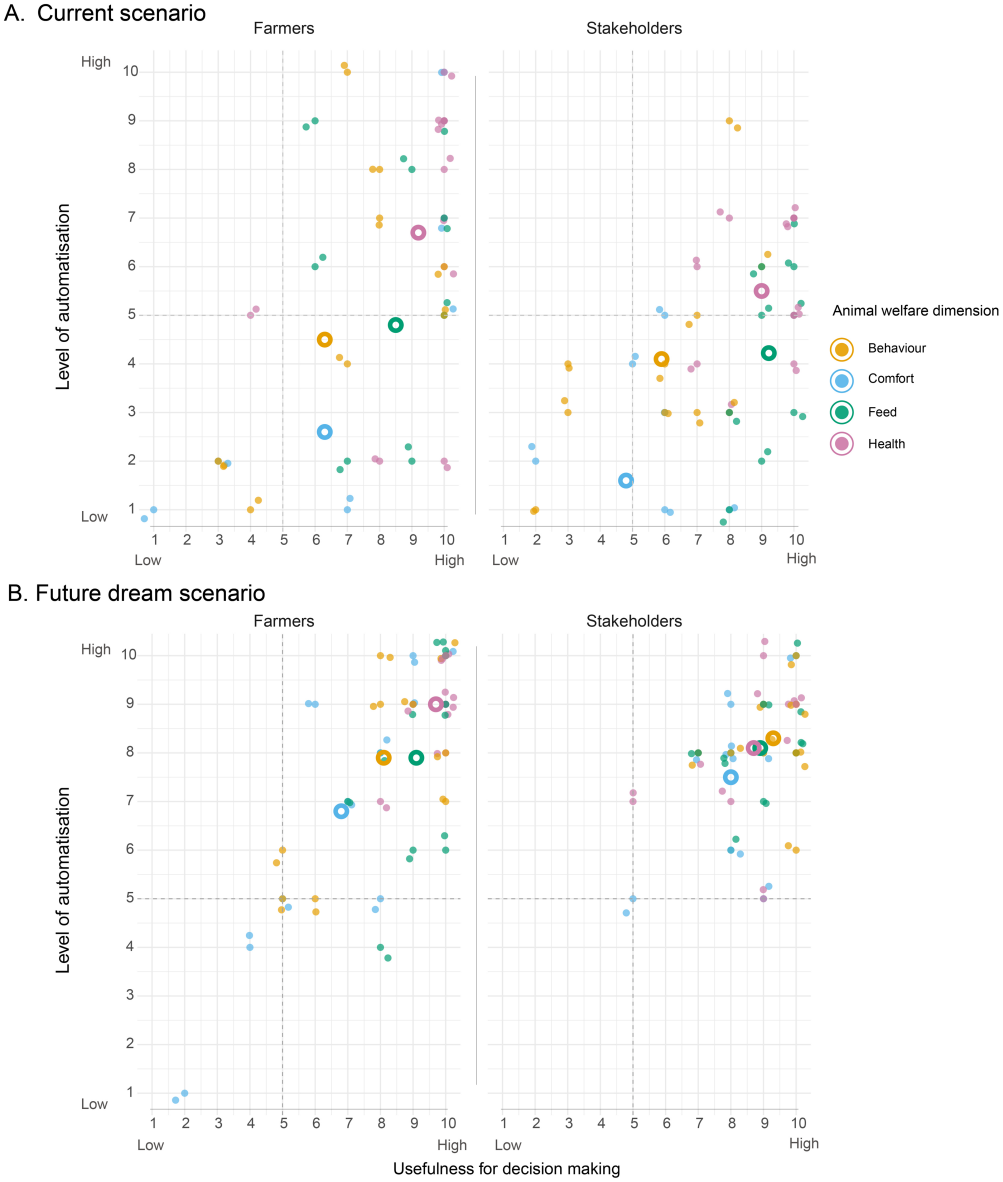
**(A,B)** Stakeholders’ (*n* = 10) and farmers’ (*n* = 10) ranking of **(A)** current level and **(B)** future dream scenario of automatisation (access to digital information/tools) and the perceived usefulness for decision-making of four welfare dimensions. Colored dots represent individual answers, while circles represent the average score of all participants. Y-axis—level of automatisation, X-axis—usefulness for decision making.

In the second focus group sessions participants were asked to rank four dimensions of animal welfare (health, feed, comfort and behaviour) according to their current level of automatisation and usefulness for decision-making. In these circumstances, health was the dimension for both farmers and stakeholders, perceived to be most automatised and useful (Figure 2A). Interestingly, the participants in both groups also agreed that the “feed” dimension was useful although with slightly lower automatisation while the behaviour and comfort dimensions were scored as less automatised and less useful in both groups. There was more variation in the farmer group regarding the current level of automatisation, likely representing farm differences. Stakeholders generally perceived a lower level of automatisation compared to the farmers, possibly reflecting that the average level of automatisation is lower in the general farm population compared to our study population.

When asked to rank the level of automation and usefulness of indicators in a future dream scenario both groups indicate a potential for increasing both the level of automation and usefulness of the indicators across all dimensions (Figure 2B). However, the dimension comfort, and to some extent behaviour, were scored as slightly less useful by farmers in comparison to stakeholders. In the discussion, all participants recognised the importance of comfort but acknowledged that it is slow to change and complex to address. Comfort was consistently perceived as hard to act on quickly because it often requires structural interventions (cubicles, mattresses, cow flow, flooring), sometimes with unforeseen consequences that impacted welfare in other ways than those intended to be solved (e.g., laying/standing disturbances due to novelty). In addition, discussions pointed to a perceived lack of good measurements for comfort. This helps explain why comfort, despite its recognised value, was relatively under-weighted in usefulness and level of automation, both currently and in a future scenario.

*“When they changed the cubicles, the milkings and yields went up within days… cows weren’t standing, avoiding lying.”* (Stakeholder, FG07)

*“Get good lying times and you might get more chafing… then rethink neck rail, mattress and shavings, solve one problem, risk another.”* (Farmer, FG04)

*“We don’t have anything that automatically indicates that cow comfort is good.”* (Farmer, FG04)

Also, awareness or detection of deviations is not enough to improve welfare. In discussion, farmers repeatedly underlined that while more automation can save labour and speed detection of deviations, decisions still hinge on stockmanship and context. Meaning that the detection of events/deviations will not improve decision quality by itself; interpretation and action remain manual tasks perceived as difficult to automate.

*“Nothing happens automatically… it has to be done manually.”* (Farmer, FG-4)

*“When there’s a health alarm, you go and check. But the alarm could be lameness, a wound, or anything—you sort it out on-site.”* (Farmer, FG-5)

*“… that final gut feeling will be hard to automate.”* (Farmer, FG-4)

Stakeholders, by contrast, were more likely to assume that richer data streams will translate into better decisions. Often pointing to a system-level combination of indicators or opportunities for benchmarking across farms. However, they also indicated that the currently available tools may not be there yet.

*“They can use it [referring to welfare indicators] to take concrete actions in their routines […] better identification and management of lame cows […] More bedding, better lying surfaces. There’s a ton to work through.”* (Stakeholder, FG-2)

*“And what I'm trying to say is like, we want to have these actionable insights, but then we always start with and maybe a step a little bit further. But I don't think we're there yet to say, yes, don't worry, farmer, we will tell you what to do. We still rely on the farmer to do the thinking and the decision, right? And we try to get closer to the actionable one, but, yeah, we are not there yet.”* (Stakeholder, FG-07)

*“I don’t think that such an automatic assessment can draw conclusions on what to work with. Like when you do a herd health plan, it can’t decide that this is what you should work with. Here, human interpretation and dialogue are necessary. Because the system does not know what happens in other parts. Nor understand the vision and motivation of humans.”* (Stakeholder, FG-2)

Farmers tend to view increased automation as labour-saving rather than decision-enhancing unless paired with interpretation and infrastructural capacity. Stakeholders, in contrast, typically expect decision value to rise with automation. The current dominance of health in the identification of sick or deviating cows reflects detection, not prevention, of disease. The minor role of comfort reflects the difficulty of achieving structural change, measurement gaps, and the difficulty of defining and enacting optimal decisions in favour of animal welfare.

### 3.3 Theme 3: Does automatisation of welfare assessment reduce “farm blindness” and change decision-making?

Participants agreed that a “future dream” of welfare assessment includes more automation of welfare dimensions. Both farmers and stakeholders valued automation for avoiding “home/barn blindness” by keeping eyes fresh and countering drift in standards. Stakeholders often framed this as resetting what “normal” means through benchmarking while farmers welcomed external eyes to challenge routine blind spots.

*“Automation can shift the normalisation scale… benchmark against lots of data to see where the best are.”* (Stakeholder, FG-2)

*“You try to read the animal… But it’s often useful to bring in external eyes… You can often become barn-blind.”* (Farmer, FG-1)

Where the groups diverged was on the future usefulness of indicators across welfare dimensions. Farmers imagined faster detection but largely the same hierarchy of useful indicators they rely on today. Other stakeholders expected greater decision value, especially from behaviour and comfort, bringing these closer to health and feeding in perceived usefulness (Figure 2). In other words, stakeholders read automation as a potential decision uplift, whereas farmers read it as vigilance uplift.

*“… most farmers would think it's a good help [referring to current tools] to make the work more effective, to find the right cows, to put the time on… usually we say like 20% of the cows should take 80% of the time, like the ones that really need attention shouldn't be that many, but you do have to put in some effort to take care of them. And this [automated assessment] would hopefully help them to put in the right care for those cows, but also like make it easier to make a decision that will make a bigger improvement for the whole herd … to do something about this [hock health] because now 60% of the animals are affected or so. Maybe you just haven't thought about it before, that it was this much or so.”* (Stakeholder, FG-7)

Farmers agreed that comfort/behaviour indicators are important, but stressed that, compared to health, these signals are too slow and complex to use for daily decisions. For example, they often point to the need for structural change but such changes go more slowly and with trade-offs. However, they highlighted that tools that would make such information more actionable in practice would be interesting.

*Those decisions are very fast [referring to health]. Or they need to be fast. And that may be the easiest to automate. […] Housing and comfort. […] What could you automate there? I don’t know how it would influence daily decisions either.* (Farmer, FG-3)

*"This use of cubicles and rising behaviour… It's not something we use all the time. At least not us."* (Farmer, FG-3)

*“Housing and cow comfort. It doesn’t change very quickly. But at the same time it would be interesting to know. Know how the cows are, where they are doing well and where they like to be. I miss that a little.”* (Farmer FG-3)

While farmers focused on the benefits of improved deviation detection, stakeholders underscored a detection–prevention gap. They emphasized that the real potential in automated welfare assessment lies in acting even earlier, using combined indicators to prevent problems before they occur.

*“We talk a lot about finding sick animals—that’s the easy part. But it’s already too late.”* (Stakeholder, FG-2)

Although farmers recognised the idea of combining information, they did not discuss it in terms of preventing future problems or alleviating current negative situations. Some also noted that while they have access to more information, which is not typically integrated into their decision-making habits and pointed to practical barriers, such as financial challenges.

*“Why couldn’t the robot combine BCS, milk, movement, eating time, cleanliness… better than any stockperson? It’s a matter of money.”* (Farmer, FG-4)

*“Those modules probably don’t show first on the computer… we find most things via rumination… I haven’t needed the queue-to-robot module.”* (Farmer, FG-6)

Yet, combining more data will not solve everything. A particular challenge, raised by both stakeholders and farmers, was the difficulty of adapting the integrated output of an automated system to different farm-specific contexts, particularly translating the output into meaningful action. Farmers repeatedly emphasised the need for trusted, long-term support to turn outputs into farm-specific plans. Stakeholders explicitly referenced this missing roadmap between more data and meaningful action, recognising that the development is slow.

*“If we keep adding more data, what will the farmer do with it? … Connecting the dots—from drinking to welfare to milk—doesn’t come from the market; it needs long-term road-mapping and research. That moves slower—no one is banging on the door for that like they are for better heat detection.”* (Stakeholder, FG-7)

These perspectives illustrate that stakeholders recognise a lag in how welfare indicators translate into meaningful action. Thus, the challenge for improving the usefulness of welfare assessments lies not in generating more data, but in connecting the dots, meaning understanding how a signal relates to welfare outcomes and what practical steps should be taken. In addition, conversations suggest a lack of clarity about who should take responsibility for this translation: the farmer, the advisor, the technology provider, or the broader industry? Thus, there is no shared roadmap, advanced functions remain underused, and the burden of interpretation falls back on farmers, who are left to grasp the system on their own. Therefore, the “advanced potential” of these systems remains largely invisible on-farm.

### 3.4 Theme 4: Split views on the value of animal welfare assessment

This theme is rooted in the differences between farmers and stakeholders regarding the practical usefulness of automation and the perceived value of animal welfare assessments for different actors across the dairy chain. In the third session, participants discussed how data from automated welfare systems should be used and by whom. While other stakeholders saw additional value in cross-farm benchmarking and programme evaluation, farmers prioritised farm-specific decision support. They cautioned that broader sharing only adds value when it tangibly improves on-farm management.

**Figure 3:**
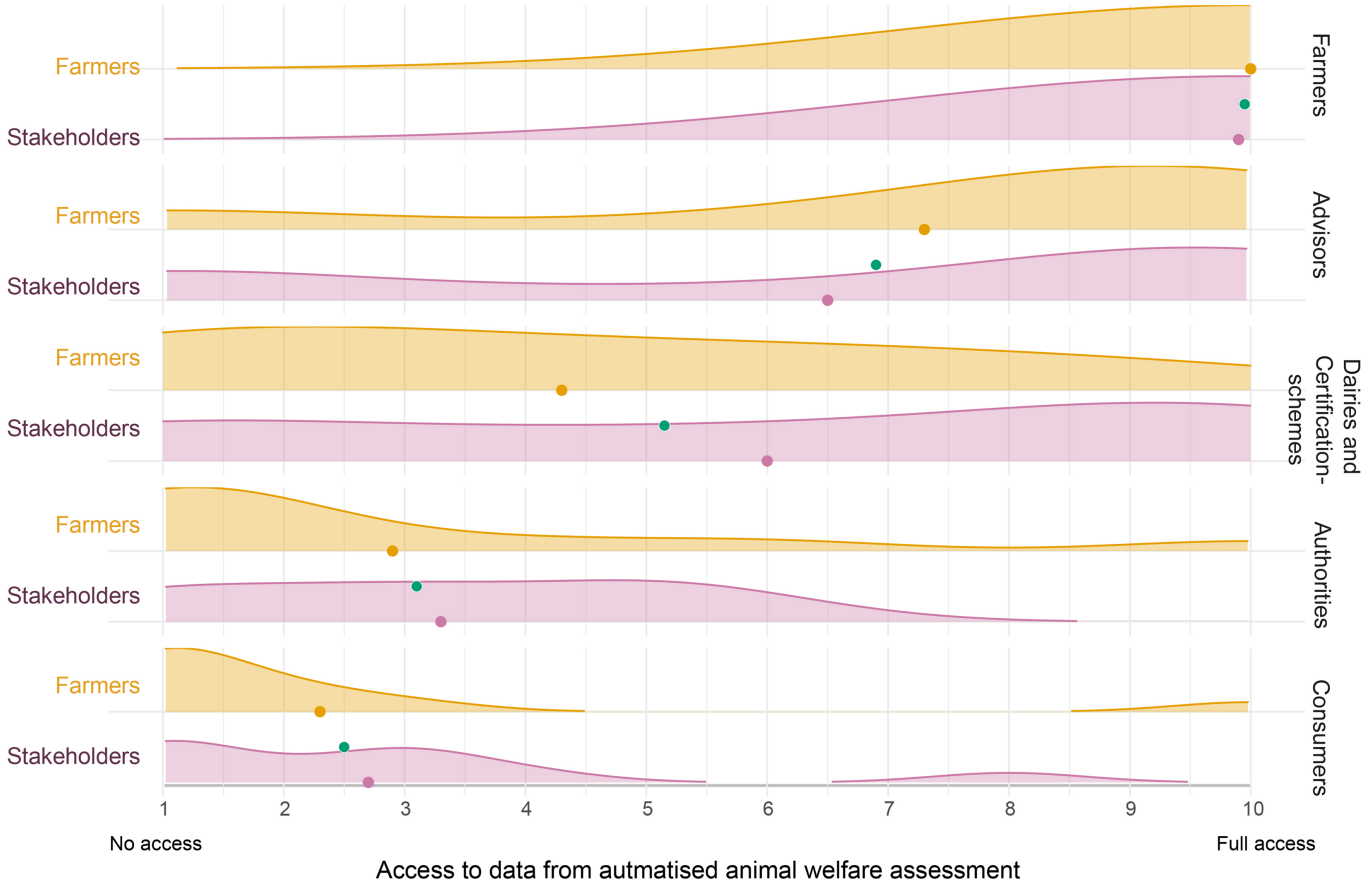
Ridge plot showing score distributions for providing access to data from automated animal welfare assessments to different groups (farmer, advisors, dairies/certifications schemes, authorities, consumers) access to data from an automated animal welfare assessment. Separate ridges represent farmers (*n* = 10, orange) and other stakeholders (*n* = 10, purple). The colored dots mark group means; the green dot represents the overall mean.

However, there was a consensus that farmers should have full access (Figure 3), reflecting a shared view that the primary value of welfare data lies on the farm, supporting day-to-day monitoring, follow-up, and timely action. Participants also stressed that farmers own the data and should control access.

*“Well, that’s pretty easy to answer. It’s the farmer who owns this data. It is up to the farmer to decide with whom to share it. That’s part of the foundation for everything we do. […] If we want to share, there should be written consent that I share with the advisor, dairy contractors or authorities.”* (Farmer, FG01).

Beyond farmer use, views diverged more, although they followed the same general trends where both stakeholders and farmers noted that advisors will benefit from having access to animal welfare data (Figure 3). The need for access from “Dairies/Certification bodies” and “Authorities” was lower, and the group “Consumers” scored lowest. Farmers’ distributions skew higher than stakeholders for their own use and advisory support, and lower than stakeholders for authorities/certification and consumers.

Farmers described a limited value in communicating data beyond sharing it with a trusted advisor, described as a person who can help turn outputs into farm-specific actions.

“*Of course they would have access to everything [referring to advisors and full data access]. Otherwise you don’t utilise most of the advisory services. And you should have complete trust in the people you work with*”. (Farmer, FG-1)

By contrast, stakeholders saw greater value in benchmarking across farms and using aggregate outputs for programme evaluation and norm-shifting. Stakeholders on the other hand saw more promise in cross-farm comparison to raise awareness and shift norms, a view already evident in Theme 3.

*“Maybe it might be better to have it at the population level or on some larger level, so that it can be used in advising and goal-setting from a broader perspective.”* (Stakeholder, FG-2)

*"Cleanliness may be more useful at higher organisational levels… moving from reactive to proactive… finding animals before they get sick."* (Stakeholder, FG-2)

While farmers also saw value in benchmarking and were interested in knowing where they stood compared to others, their own-farm trajectories mattered more. So, rather than a between-farm comparison, their focus was on the potential of the tools in helping them judge whether their own management is working. They mention that herds, buildings, and routines are unique and expressed difficulty in learning from farm comparisons since each farm has its own challenges, explaining why between-farm comparisons felt less relevant.

“*Yes, but if you don’t have the interest, then it’s hard to see… well, where you stand… or what use you really have for the information you get. You have to be able to connect it to reality, so to speak. Otherwise it’s just like, yeah, I get all these numbers but what do they mean? And then you have to be able to adapt the system to your own farm. Which information is most useful to us, and how should we use it? It’s… it’s mostly… well, you have to think it’s fun to sit and connect things and tinker with it, I think. Otherwise it won’t get done either.*” (Farmer, FG-6)

“*Well, comparing farms would be absolutely great fun, but usually that’s all it is — just comparing other farms with different conditions, other… different farms are financed in another way, and then it’s very hard for some who have a bit less money in the budget to compare themselves with those who have much more. But sometimes you can feel very satisfied when you manage to keep up. But whether it’s always a good thing, I don’t know. Sometimes you can feel very sad that you don’t reach your goals.”* (Farmer, FG-1)

Another recurring concern was the risk of misinterpretation when sharing animal welfare assessments beyond the farm, particularly with audiences perceived as distant from practice (authorities, consumers). The views on sharing data with dairy companies and certification schemes varied across the scale and farmers voiced perceived benefits such as better prices for products, transparency, simplified workload and objective assessments. However, they also worried about data sharing becoming a mandatory “cost” without benefit and that data would be assessed out of context and without understanding and dialogue. Thus, they saw opportunities for support but were afraid that it would not be used in a way that benefited them.

“W*e work to create and handle this data [referring to welfare indicators]. In the end we should get paid for the product we deliver and so on. This type of data can increase product value. Then we shouldn’t let it go without bargaining about it.*” (Farmer, FG-1)

*“No, but I think it’s very connected — the more information the dairy is supposed to have, the better the communication and dialogue have to be. Otherwise, I think it just gets really strange.”* (Farmer, FG-4)

A fear of sharing data was also expressed for authorities as well as consumers. The dominant view was that context gets lost, leading to misunderstandings that would reflect negatively on the farm. In addition, both farmers and stakeholders stressed that any information shared with consumers would need careful translation and a clear benefit to the farm, not only to others.

*“They [the county administrative board] are supposed to be on our side and help us. But they’re out looking for faults, and then of course you also want to hide everything. Unfortunately, that’s probably an image that reflects much of the country — at least that’s how we experience it.”* (Farmer, FG-4)

*"I have many other visitors… often uninformed about animals and production—communicating with consumers is challenging."* (Farmer, FG-1)

*“There has to be a discussion about the statistics or the underlying material. That’s where I have some concerns about just giving it out. And then you have people who know nothing about cows making judgments about something they don’t understand.”* (Farmer, FG08)

Combining the ideas developed into themes indicates that all participants agree on the importance of pursuing good animal welfare regardless of their work. Automation of welfare assessments is an avenue for farmers to improve efficiency in flagging issues. However, any information sharing beyond the farm depends on trusted relationships and supports that carry signals further than dashboards and ensure benefit-sharing aligned with farm goals.

## 4 Discussion

Our paper aimed to understand the tensions around a potential automated welfare assessment tool across the dairy system. By contrasting the farmer and other stakeholder perspectives on existing digital tools, their decision-making and views on data sharing, we tested if, where and how further automation of animal welfare assessment adds value. Our analysis developed four themes that, collectively, show that values are aligned while the logics of use diverge. Farmers and other stakeholders agree that animal welfare matters and should be improved continuously, yet they attach different expectations to automation. Farmers describe automated welfare indicators primarily as surveillance tools that can be used to create lists and alarms that make them faster at noticing deviations, while stakeholders more often assume that additional data will directly improve decisions and enable system-level evaluation. Read against our first and third themes, along with the ranking results, the divergence between stakeholders and farmers is not rooted in different perceptions or values regarding animal welfare, but different views on the steps needed to go from signal to change in a specific farm context.

The current focus on detecting deviations suggests that the currently available indicators are underutilised when it comes to supporting change. The COM-B method, a behaviour and implementation theory (16), helps clarify why alerts rarely shift practice. COM-B is a behavioural diagnosis method stating that Behaviour (B) occurs when Capability (C), Opportunity (O), and Motivation (M) are sufficient, so behaviour change is achieved by targeting one or more of C, O, and M. Our conversations highlight that while automation increases capability with timely cues, and at times motivation through feedback; opportunity expressed as time, labour, capital, and feasible system/production options—often constrains follow-through. This was especially true for comfort/behaviour aspects where gains were mentioned to require structural changes (cubicles, mattresses, flooring) and risk trade-offs. The focus group conversations convey that this is the challenging part as each farm is unique. Thus, actions need to be farm-specific even when indicators are standardised and communication moves from “What’s the problem?” to “How will we solve it here?”

Yet, data usage and sharing between farmers and stakeholders beyond trusted farm advisors often focuses on a reflexive monitoring (benchmarking), although it is known that audit-and-feedback in the form of information alone has limited effects (24, 25). The normalization process theory (13) explains that for practices to be implemented, embedded and sustained, four mechanisms must be present and shared across all stakeholders. These include coherence (sense making), cognitive participation (engagement of all parts involved), collective action (enacting), and reflexive monitoring (appraisal). Our results indicate that although the four mechanisms may be present, their value is not shared among farmers and stakeholders. Stakeholders highlighted a joint struggle and need to increase monitoring and go deeper into the available information to support the best animal welfare in a long-term perspective. Farmers on the other hand emphasised the need to make sense of animal welfare indicators within their farm context (coherence) as well as a need to know how to improve welfare, avoiding trade-offs, on their farm. However, the collective action in the form of feedback coupled with explicit shared goals, support and action plans, that could most likely trigger change in on-farm behaviour, was missing. This supports the same conclusion as the participating farmers articulated: automation is necessary for awareness but insufficient for change.

Health cues (e.g., drops in rumination/eating, mastitis indicators) dominated the current usability of automation while indicators of animal comfort lagged (Themes 1–2). Health cues point to near-term, controllable actions that a farmer can isolate and adjust, where automation adds tangible value by speeding detection (1, 26–28). Gains in comfort or gains in normal or positive behaviour are slower, for example given its potential dependence on infrastructure and improvements in overall husbandry design, at times with uncertain payback and consequences as described in Theme 2. Also, changing stall surface and maintenance to meaningfully affect lying time, lameness risk, and preferences often requires renewed infrastructure and time investment along with capital (14, 29–32). Thus, farmers express that they lack tools as well as an understanding of economic consequences (economic losses, and required investments matter), which in turn will impact their perceived ability to create change (33–35). Similar reasoning has previously been suggested for lameness where the costs are substantial, but paybacks are distributed and delayed, underscoring the need for financing, staged plans rather than dashboards of data or alerts alone (36). Our results provide evidence that the perceived benefits and constant cues to action, and salient barriers (time, cost, disruption) are different across animal welfare aspects. To handle more complex welfare challenges tools or education alone is not enough but workable rules and processes that protect the local context are also needed, in combination with economic sustainability (9, 13, 35).

Theme 3 developed the idea of a detection–prevention gap. In the future, stakeholders expected gains in comfort/behaviour from more automation and combined signals, while farmers saw vigilance uplift, faster detection with roughly the same hierarchy of useful indicators. Both positions are reasonable as sensor validation continues to advance and include more parameters, yet integration for preventive decisions remain a work-in-progress, particularly on pasture and for higher-order welfare constructs (1, 26–28, 37–39). The automation-bias literature offers a useful caution: more data can amplify noticing without ensuring doing unless systems are designed to support the right next step (40, 41). Our participants asked for exactly that: people who can ‘read’ the tools, plus advisory continuity, financing, and stepwise plans that translate outputs into farm-specific actions (Theme 3). If this is not put in place there is a risk that the potential of automated animal welfare indicators is not used to their full potential.

Theme 4 extended the argument to who should see data and where value arises (Figure 3), detecting additional challenges that require addressing. The pattern of results is consistent with concerns about misinterpretation and benefits arising elsewhere than on the farm itself. Farmers emphasised that the data welfare assessments should be used for within-farm trajectories (“are we improving here?”) and were cautious about sharing for benchmarking purposes which ignores local context and, with that, feasibility. Yet, stakeholders saw much promise in benchmarking to shift norms, evaluate programmes and deepen understanding of animal welfare. These tensions mirror broader debates about data governance and trust in the dairy sector, i.e., who owns the data, who benefits, and how should the value be shared (42–45). The implication is not to avoid sharing, but to couple it to farm-level benefit in the form of advisory support that turns signals into doable plans, and feedback that provides evidence of within-farm improvement over time (24, 25, 42).

To achieve this, there are important research/practice gaps that need to be filled. First, animal welfare indicators (animal and resource based) are often used as proxies for complex welfare states. Our discussions around automation visualise that there is a lack of knowledge about how measures interact in real farms. A systems-level research agenda where interactions between indicators are linked to specific action pathways and economic consequences would better serve on-farm decision-making than isolated device validation (1, 26–28, 37–39). Second, implementation remains the missing middle: vendors and schemes tend to deliver dashboards, while farmers need dashboards with do-able plans; i.e., attention lists tied to SOP bundles (“who checks what; what counts as resolved”), templated, costed comfort retrofits, scheduled follow-ups, and proof-of-benefit feedback (13, 25, 35). In short, the sector must move from assuming that automation will drive change to designing for change.

Certain methodological and practical limitations must be acknowledged to contextualise our findings. This study is exploratory and grounded in the Swedish dairy context, which may influence how the findings apply to other production systems. We acknowledge that most farmers who participated might already be interested in animal welfare and digital innovation, which may have shaped the perspectives captured. However, it can also enrich captured perspectives, which aligns with the qualitative aim of engaging information-rich participants to explore meanings in depth. The online format, may have limited the richness of interaction between participants and researchers, making it harder to observe non-verbal cues. Misinterpretations between participants could be present due to audio or lag effects, thus influencing participation. Still, Mentimeter facilitated engagement and allowed participants to express their views non-verbally. This was confirmed by the aftermath comments by participants who found the sessions engaging. On the positive side, the online format enabled participation from geographically dispersed stakeholders as well as group continuity, creating opportunity for social interaction, trust and iterative reflection across sessions.

## 5 Conclusion

Our study shows that farmers and stakeholders share norms and goals for animal welfare yet diverge in how automated indicators are used and where the benefits land. These is explained through four interlinked themes, including (1) alignment in welfare values but divergence in indicators’ daily usefulness; (2) structural and contextual barriers to achieving the ‘best welfare’; (3) a detection–prevention gap in automation; and (4) differing views on data ownership and value distribution. For farmers, automation is necessary for awareness but currently insufficient for change: it mainly scales detection and targeting of sick or deviating animals, so its decision value remains low unless a farm-specific strategy turns signals into feasible actions. Stakeholders, by contrast, anticipate decision uplift, primarily through benchmarking and combined indicators, but this potential is not visible at the barn door without bridges that translate data into doable plans.

The tension is sharpest for comfort/behaviour, where meaningful gains require structural changes (housing, flooring, cow flow) with slow payback; here, more data alone does not move decisions. Bridging the gap means shifting advisory encounters from problem-listing to co-planning, and pairing data with the economic and organisational scaffolds that make change feasible: attention lists linked to ready-to-use SOPs, phased and financed comfort improvements, and trusted advisory continuity (including peer support), and feedback on within-farm improvement over time.

Beyond the farm, value depends on trust, translation, and benefit-sharing; otherwise, sharing risks misinterpretation while benefits accrue elsewhere. As a qualitative study in the Swedish context, our findings call for implementation trials that test dashboards with action pathways and financing, and for governance models that align data use with on-farm benefit. In short, automation raises alertness. Yet, to achieve meaningful change, data must be coupled with farm-specific plans, financing, and trusted support.

The practical implications of our study call for technology designers to prioritise usability and decision relevance over data quantity, as well as the integration of advisory feedback loops and farm-specific planning functions. Efforts should also include support from governance bodies and welfare schemes to farmers in a long-term advisory continuity, and the development of data governance frameworks that reward farmers for welfare transparency while ensuring contextual interpretation.

## Data Availability

Supplementary material, including the complete Mentimeter question sets and focus group script is archived together with the anonymous data (i.e. results) from the farm rankings conducted during the focus groups. All materials are available in the Open Science Framework (OSF) at doi: 10.17605/OSF.IO/8EF5A. Due to privacy considerations, full interview transcripts will not be shared, as participants come from a small dairy community in Sweden and may risk identification. However, a summary of relevant interview quotes can be made available from the corresponding author upon reasonable request.

## Footnotes

^1^ https://osf.io/8ef5a/overview
